# Rapid Fabrication of Microporous BaTiO_3_/PDMS Nanocomposites for Triboelectric Nanogenerators through One-step Microwave Irradiation

**DOI:** 10.1038/s41598-018-32609-6

**Published:** 2018-09-24

**Authors:** Shin Jang, Je Hoon Oh

**Affiliations:** Department of Mechanical Engineering, Hanyang University, Ansan, Gyeonggi-do 15588 Republic of Korea

## Abstract

Even though porous elastomers and elastomeric nanocomposites have shown many advantages for triboelectric nanogenerators (TENGs), their fabrication techniques are relatively complicated, inefficient, and time-consuming. In this work, we introduced a simple, efficient and rapid concept to fabricate porous polydimethylsiloxane (PDMS) nanocomposites. PDMS nanocomposites with various porous structure were produced within a few minutes through just one-step microwave irradiation without any post-processing. Three solvents with different boiling points were selected as sacrificial materials to control porous structure. To fabricate nanocomposites, BaTiO_3_ (BT) nanoparticles were mixed into the uncured PDMS and sacrificial solvent mixture. Additionally, Ni nanoparticles were also used to understand the effect of embedded material’s property on porous structure. The porous BT/PDMS nanocomposites fabricated via microwave irradiation greatly enhanced the electrical performance of TENGs as compared to a pure solid elastomer. The present study provides a simple, rapid and inexpensive approach for fabricating TENGs based on porous elastomeric nanocomposites.

## Introduction

Energy harvesting from ambient renewable resources is of interest nowadays due to energy problems. The triboelectric nanogenerator (TENG) has emerged as a particularly promising energy harvesting device because it has a high electric potential output and simple structure compared to other devices such as piezoelectric or pyroelectric nanogenerators^1–4^. With further improvements in the electric output and fabrication process of TENGs, it could have even more advantages^5–15^.

Polydimethylsiloxane (PDMS) elastomers have been widely used in TENGs due to its good triboelectric properties, superior flexibility and stretchability, and non-toxicity, as well as its ability to be easily modified by constructing various structures in the bulk or on the surface^16–19^. Recently, porous PDMS has been found to have better triboelectric properties than solid PDMS. If metallic/organic/inorganic materials are used to produce PDMS composites with a porous structure, these materials would have many advantages in TENG^20^. To produce a porous structure, there are several fabrication methodologies, including direct-template, emulsion-template, gas-foaming, phase-separation, or 3D printing techniques^21^. Among these, the direct-template method has been widely used for TENG fabrication because it enables control of pore size and porosity. Kim *et al*. fabricated sugar templates as a sacrificial material, then immersed the template in uncured PDMS^22^. The PDMS base on the sugar template was then cured and the template was washed away. Lee *et al*. also developed a porous PDMS film using sacrificial polystyrene spheres^18^. The polystyrene/PDMS mixture was cured at 90 °C before being soaked in acetone for 24 hours. For the porous composites, Fan *et al*. adopted the similar approach to fabricate porous CNT/PDMS composites using NaCl powders. However, such methods for fabricating porous PDMS and PDMS composites involve independent curing and etching processes, each of which requires a long time because a conventional heating process is used. This is inefficient in terms of the fabrication cost and material usage. Therefore, a novel methodology that is fast, efficient, and easy to fabricate would have many advantages.

Microwaves are a form of electromagnetic waves with wavelengths ranging from one millimetre to one meter and frequencies between 300 MHz and 300 GHz. Among the several applications of microwaves, microwave (MW) with 2.45 GHz frequency has been widely used for heating. The MW heating process is basically different from the conventional (direct) heating process using a hot plate or a furnace. During MW irradiation, heat is generated internally within a material as opposed to originating from external heating sources. Translational motion of free or bound charges in materials is induced by MW irradiation and this kinetic energy is converted into heat. Therefore, extremely rapid, efficient and selective heating compared to direct heating is possible based on materials selection. MW irradiation has been used for synthesizing^23–26^ and curing^27–29^ organic materials (or metal nanoparticles), as well as cooking and baking. Nevertheless, there have been few investigations on the fabrication of porous polymers by MW irradiation, which is used only to induce a chemical reaction after curing the polymer film in the conventional heating process^30^. In addition, to the best of our knowledge, a methodology for using MW irradiation to make a porous structure while simultaneously curing, in particular to produce porous elastomers and porous elastomeric nanocomposites, has not been reported.

In this study, a rapid baking process through one-step microwave irradiation is introduced to directly fabricate porous PDMS nanocomposites without any post-processing. In order to construct the porous structure, deionized (DI) water or one of fluorine solvents with different boiling points is directly mixed with the PDMS precursor. Pure PDMS with specific porous structure is then obtained by MW irradiation. It is shown that pore size could be simply controlled according to the boiling point. Furthermore, it is demonstrated that various PDMS nanocomposites with porous structure can also be produced using this method by incorporating BaTiO_3_ (BT) nanoparticles. Conductive nickel nanoparticles were also used for nanocomposites to understand the effect of incorporated material’s property on porous structure. Finally, it is verified that porous BT/PDMS nanocomposites fabricated using MW irradiation greatly improve the performance of TENG. The methodology proposed in this study could be applicable to TENG as well as a variety of other applications^31–35^ where porous polymers are used.

## Methods

### Materials

PDMS (Sylgard 184, Dow Corning Co.) was purchased for fabricating porous elastomeric nanocomposites. Two fluorine solvents, EGC-1720 and FC-40, and DI water was used as a sacrificial solvent. The boiling point and surface tension of the additive solvents are summarized in Table 1. BT nanoparticles (467634, Sigma Aldrich Co.) were selected as incorporating materials because it has strong ability to improve triboelectric performance^36,37^. Additionally, nickel nanoparticles (DT-NI-S10, Ditto Technology Co.) with the similar diameter of BT nanoparticles were purchased to investigate the effect of material property on porous structure.

**Table 1:** Material property of sacrificial solvents.

Solvent	EGC-1720	DI water	FC-40
Boiling point (°C at 1 atm)	61	99.97	165
Surface tension (mN/m at 25 °C)	13.30 ± 0.13	72.41 ± 0.15	15.79 ± 0.13

### Fabrication process

**Figure 1:**
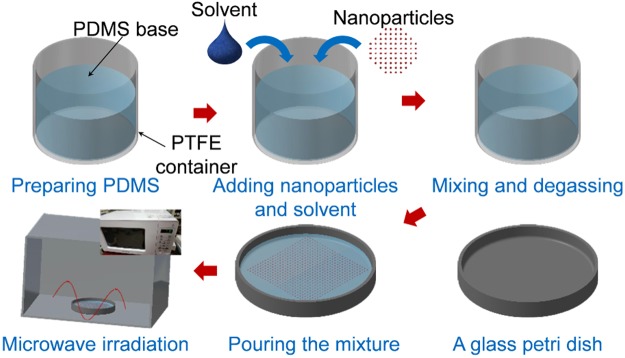
A schematic diagram of fabrication process.

Figure 1 illustrates the fabrication procedure. One of two fluorine solvents, EGC-1720 and FC-40, or DI water is manually premixed with the PDMS base and curing agent (10:1 in weight ratio). The mixture is then blended again for 3 minutes at 2000 rpm via a centrifugal mixer (ARE-310, Thinky Co.). A total of seven precursor mixtures were prepared to fabricate porous PDMS, and each was labelled for convenience depending on the volume ratio, as listed in Table 2. The precursors were degassed in vacuum for 15 min to remove residual air pores. MW irradiation was then performed on all the mixtures for 2 min 30 sec. Through the process, the mixtures were cured and porous structures were made at the same time.

**Table 2:** The mixing volume ratio of the precursors for fabricating porous PDMS.

Name of sample	PDMS base	EGC-1720	DI water	FC-40
Sample 1	1			
Sample 2	11	4		
Sample 3	11			4
Sample 4	11		4	
Sample 5	12		3	
Sample 6	13		2	
Sample 7	14		1	

### Characterization

The surface tension of the sacrificial solvents was measured using a surface tension analyzer (DST-60, SEO Co.). To visualize and measure temperature, a thermographic camera (TG-165, FLIR Co.) was used. The microstructure was characterized using field-emission scanning electron microscope (FE-SEM: S-4800, Hitachi Co.). To determine the physical form of the PDMS nanocomposites, X-ray diffraction (XRD: D/Max-2000, Rigaku Co.) analysis was performed. An in-house motorized system controlled via LabVIEW was used to test the TENGs. The contact force of the TENGs was measured using a load cell (UMM-K20, Dacell Co.). The applied contact force was recorded through a data acquisition board (PXIe-4330, National Instruments Co.) mounted on a PXI chassis with a controller (PXIe-8135 and PXIe-1082, National Instruments Co.). For all evaluation of TENGs, the applied force and frequency were fixed to 6 N and 2 Hz, respectively. An oscilloscope (MDO-3012, Tektronix Co.) and a preamplifier (SR570, Stanford Research Systems Co.) were used to measure voltage and current, respectively.

## Results and Discussion

### MW heating process and porous PDMS

**Figure 2:**
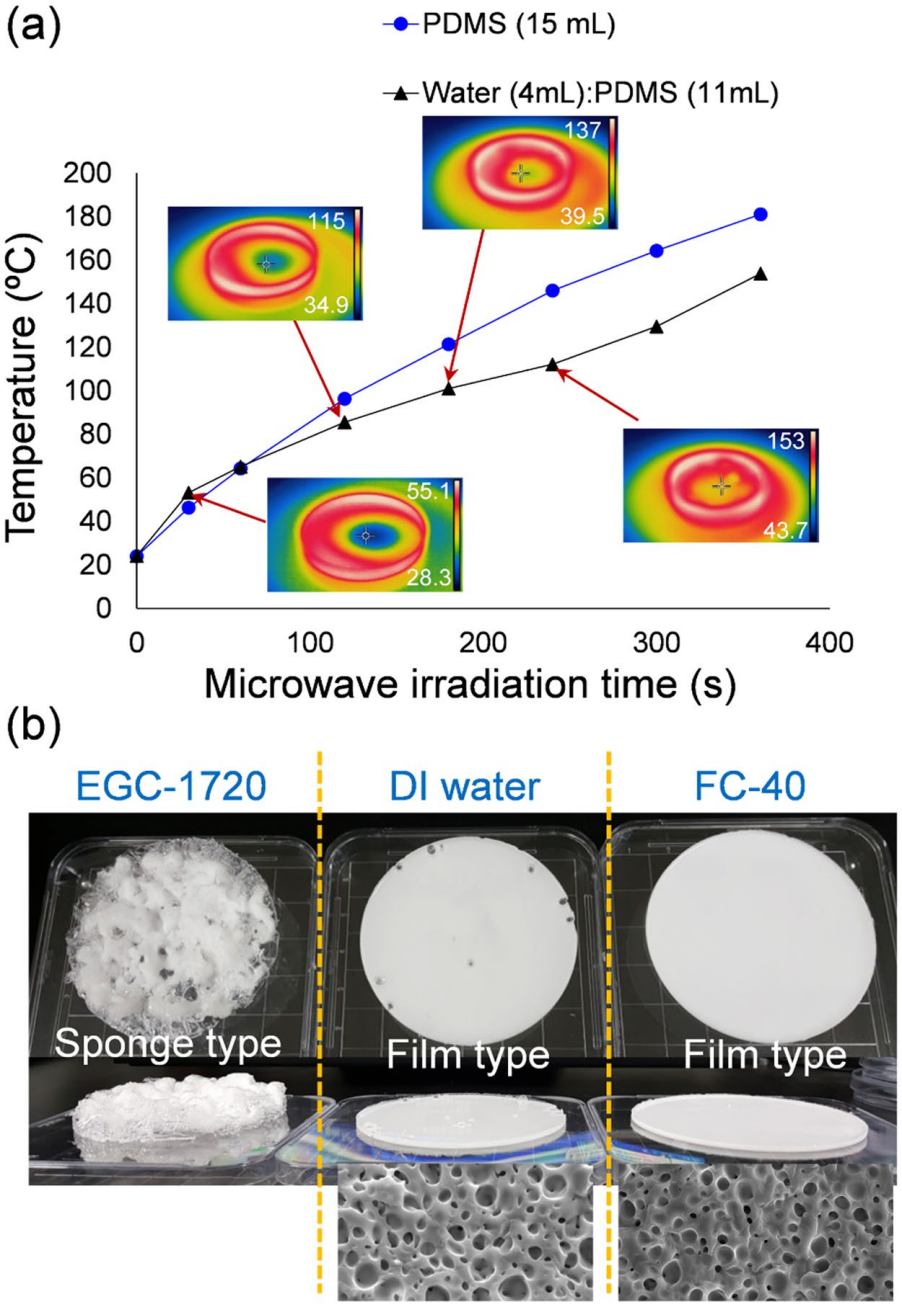
(**a**) Temperature variation of PDMS precursor during MW irradiation. (**b**) Photograph and FE-SEM images of fabricated porous PDMS.

Firstly, the temperature variation was measured with respect to MW irradiation time. The pure PDMS (Sample 1) and water/PDMS mixture (Sample 4) showed different temperature trends (Fig. 2a). The temperature logarithmically increased for Sample 1 as the PDMS cured, but when DI water was mixed with the PDMS base, an interesting temperature change was observed. The oscillation of water molecules induced rapid heating of the sample initially. As MW irradiation continued, the temperature increased slowly because the water began to evaporate. When all of the water evaporated, the temperature of the PDMS rapidly increased again. After analyzing this temperature variation, we fabricated porous PDMS using three different additive solvents—DI water, EGC-1720, and FC-40 in order to study how the different boiling points of solvent affect the porous structure. Figure 2b shows photographs of the porous PDMS and the microstructure of the cross-section of the fabricated Sample 2–4. Those three samples are obtained from the same volume (15 mL) of precursor mixtures and two types of porous PDMS are observed. When EGC-1720 was used, the PDMS was extremely swollen during MW irradiation and thus sponge-type PDMS with millimeter-scale large pores was obtained. When the sacrificial solvent was changed to DI water, swelling phenomena were significantly suppressed even though a few large pores were constructed. In the meanwhile, such large pores were not observed at all for FC40/PDMS mixture. Although they appear as solid PDMS films for both the Sample 3 and 4, cross-sectional FE-SEM images at the bottom of Fig. 2b clearly show that many micropores are well and uniformly constructed in the PDMS. This result can be explained by the boiling point of the additive solvent. EGC-1720 starts to evaporate at 61 °C, which means that a rapid transition from liquid to gas phase occurs, eventually leading to extreme expansion of the gas. It was clear that the swelling of the PDMS was suppressed as increasing the boiling point. The areas where the sacrificial solvent was distributed were rapidly replaced by air and simultaneous curing was possible. MW irradiation could rapidly construct porous structures, and it shows possibility of controlling the size of the pores by the boiling point.

Three TENGs were fabricated using Sample 1–4 to evaluate their triboelectric properties. For all TENGs, the contact area was 4 cm × 4 cm and thickness of the porous PDMS were ~ 1 mm. Due to severe swelling, Sample 2 was excluded in fabricating TENG. The TENGs were evaluated using an in-house system, as shown in Fig. S1a and Movie S1. Polyimide (PI) film was selected as a counter friction material for the porous PDMS as well as the porous PDMS nanocomposites. Based on the contact and separation of the friction materials, PI and PDMS, a graph of voltage as a function of time was obtained (Fig. S1b). In this study, all of the TENGs were operated in contact-separation mode^4^. There were no charges in the materials initially (Fig. S2-i). When two friction materials come into contact, one material loses its electrons and the other gains electrons. In this process, all charges generated on both surfaces are ideally in the same plane (Fig. S2-ii). As the materials separate (releasing and fully released), the electrical equilibrium is broken, allowing the free electrons in the electrodes to move to balance the broken equilibrium (Fig. S2-iii and S2-iv). When the materials are pressed into contact again, the free electrons return to their original position (Fig. S2-v). The voltage or current was obtained depending on the electrical wiring of a load resistor between two electrodes. When the average positive/negative and peak-to-peak voltages were compared (Fig. S3), the TENG based on porous PDMS fabricated using DI water or FC-40 exhibited a greatly enhanced output compared to that based on pure solid PDMS (Sample 1). Since the TENG based on Sample 4 had the highest voltage output of the three porous PDMS samples, DI water was selected for fabricating porous structures in the rest of this study.

**Figure 3:**
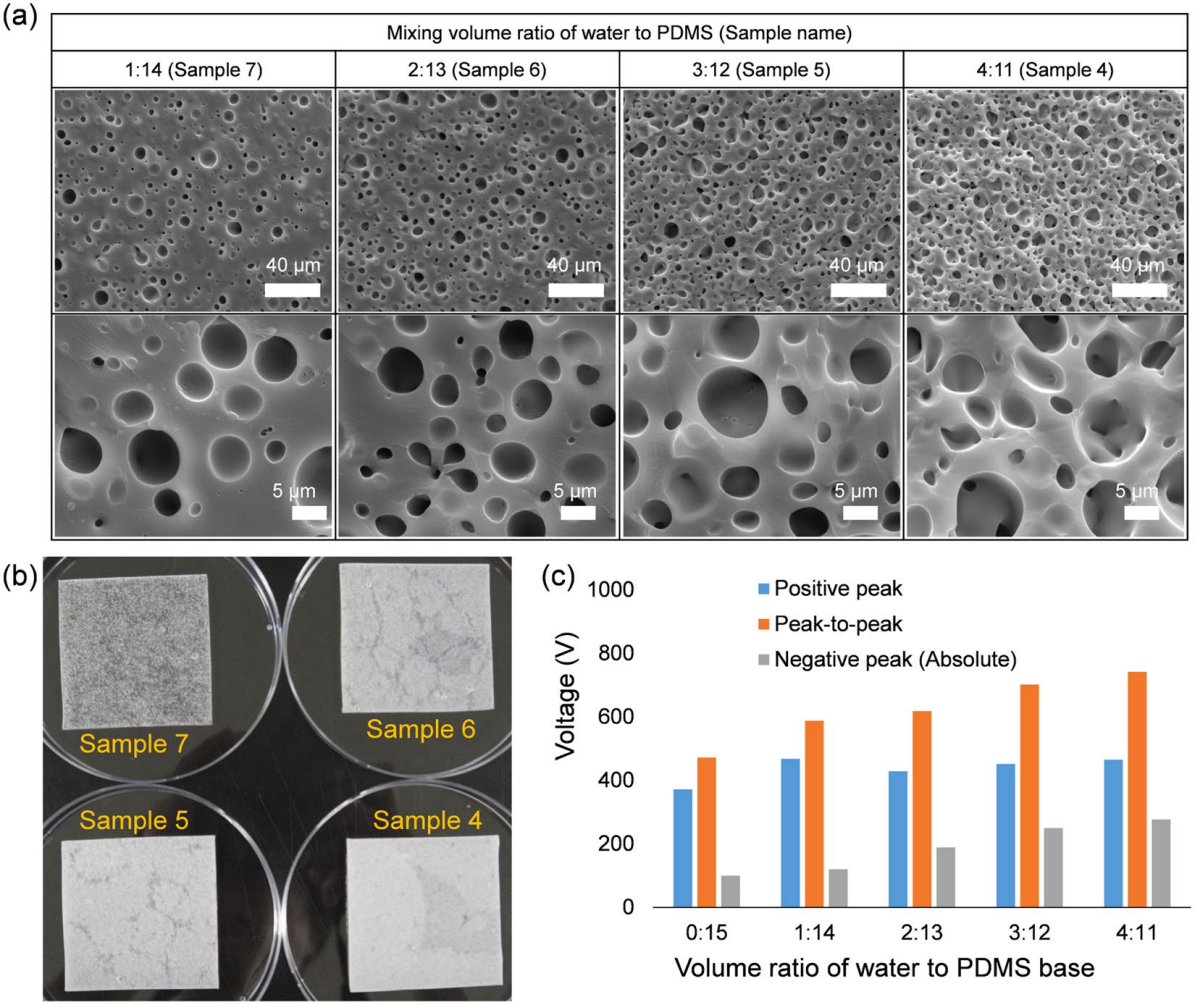
(**a**) Cross-sectional FE-SEM images of porous PDMS fabricated from water and PDMS mixture as a function of the volume ratio. (**b**) Photographs of porous PDMS fabricated from the water/PDMS mixture with respect to volume ratio. (**c**) Comparison of the voltage output of TENGs based on porous PDMS fabricated via MW irradiation.

We further investigated the effect of volume ratio on both the porous structure and the performance of TENG. Figure 3a,b respectively show cross-sectional FE-SEM images and photographs of the porous PDMS as a function of the volume ratio of DI water to PDMS base. Many air pores ranging from 1 μm to 20 μm are observed and well distributed inside the PDMS. As expected, the greater the volume ratio of DI water, the more air pores are generated. Volume ratios of up to 4:11 were studied; as the volume ratio of water increased further, it became difficult to mix the DI water and PDMS. It should be noted that although the volume ratio of DI water can be increased, the present study has focused on the MW irradiation process instead of preparation of a precursor. When used in TENGs, the peak-to-peak voltage output gradually increased with increasing volume ratio (see Fig. 3c). When it comes to the performance of TENGs, it is important to increase electric charges generated on its surface. Fig. S4 illustrates basic structure of a contact-separation mode TENG. At open-circuit condition, the output voltage of TENG is defined as follows^38^:

1 $${\rm{V}}=\frac{{\rho }_{A}\cdot d(t)}{{\varepsilon }_{0}}-\frac{Q}{{\varepsilon }_{0}}(\frac{{d}_{1}}{{\varepsilon }_{r1}}+\frac{{d}_{2}}{{\varepsilon }_{r2}}+d(t))$$

where *ρ*_*A*_, *Q*, *ε*_*r*1_, and *ε*_*r*2_ are the charge density of the contact surface, transferred charges, and relative permittivity of each material, respectively. The *d* denotes thickness of a material. From equation (1), it has been well known that the amount of charges generated in the materials, dielectric property and thickness are really important in terms of the electrical performance. At the open-circuit (OC) condition, *Q* becomes zero because there is no chrage transfer. Thus, the OC voltage (*V*_*OC*_) is given by

2 $${V}_{OC}=\frac{{\rho }_{A}\cdot d(t)}{{\varepsilon }_{0}}$$

In equation (2), the *ρ*_*A*_ is proportional to the capacitance of the TENG at the OC condition. The capacitance is proportional to ${\varepsilon }_{r}/{d}_{PDMS}$ and porous structure is more deformable, which enables to decrease the effective thickness$({d}_{PDMS})$ more readily, so more charges are generated with porous structure. Moreover, the porous structure could increase the amount of charge generated in the material due to its enlarged surface area. Thus, the output voltage gradually increased with the increase of porosity^39^. Interestingly, the porous PDMS fabricated with DI water had a significantly greater increase in negative voltage than in positive. That is, the output increases more when the porous PDMS and the PI film contact each other. Compared to pure solid PDMS, the positive and negative voltages increased by over 25% and 170%, respectively.

### Solid PDMS nanocomposites

**Figure 4:**
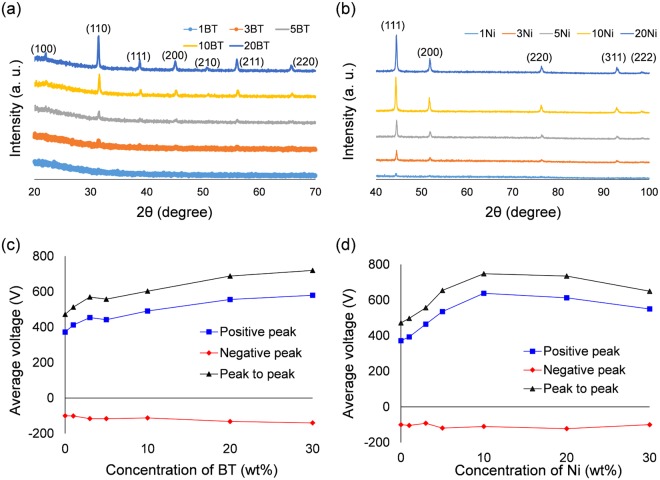
XRD patterns of (**a**) BT/PDMS and (**b**) Ni/PDMS. Average voltage output of TENGs fabricated using (**c**) BT/PDMS and (**d**) Ni/PDMS nanocomposites.

Prior to studying porous PDMS nanocomposites, solid PDMS nanocomposites were fabricated by curing the BT (or Ni) and PDMS precursor. A total of ten samples were fabricated depending on concentration, and the samples were labelled as listed in Table 3. MW irradiation was able to easily cure BT/PDMS nanocomposites without any phenomena such as swelling (Fig. S5). There was no significant difference between BT/PDMS and Ni/PDMS nanocomposites although electrical properties of BT and Ni are different (Fig. S6). As the concentration of nanoparticles increased, more particles were observed in the FE-SEM images (Figs S7 and S8). Figure 4a,b show the XRD patterns for both nanocomposites with respect to the concentration of BT and Ni. When the concentration was less than 5 wt%, few or no peaks were observed. As the concentration increased above 10 wt%, the peaks appeared at the same 2 theta angles as in the XRD patterns of pure BT and Ni^40,41^. Similar to the porous pure PDMS, when the TENG fabricated using Ni/PDMS and BT/PDMS nanocomposites is tested, the overall voltages including positive/negative and peak-to-peak ones were increased (Fig. 4c,d). When the concentration is more than 10 wt%, the peak becomes clear in XRD patterns, which is consistent with the improvement tendency in TENG.

**Table 3:** Labelling of BT/PDMS and Ni/PDMS precursors depending on concentration.

Name of sample	Concentration (wt%)	Name of sample	Concentration (wt%)
1BT	1	1Ni	1
3BT	3	3Ni	3
5BT	5	5Ni	5
10BT	10	10Ni	10
20BT	20	20Ni	20

### TENG based on porous PDMS nanocomposites

**Figure 5:**
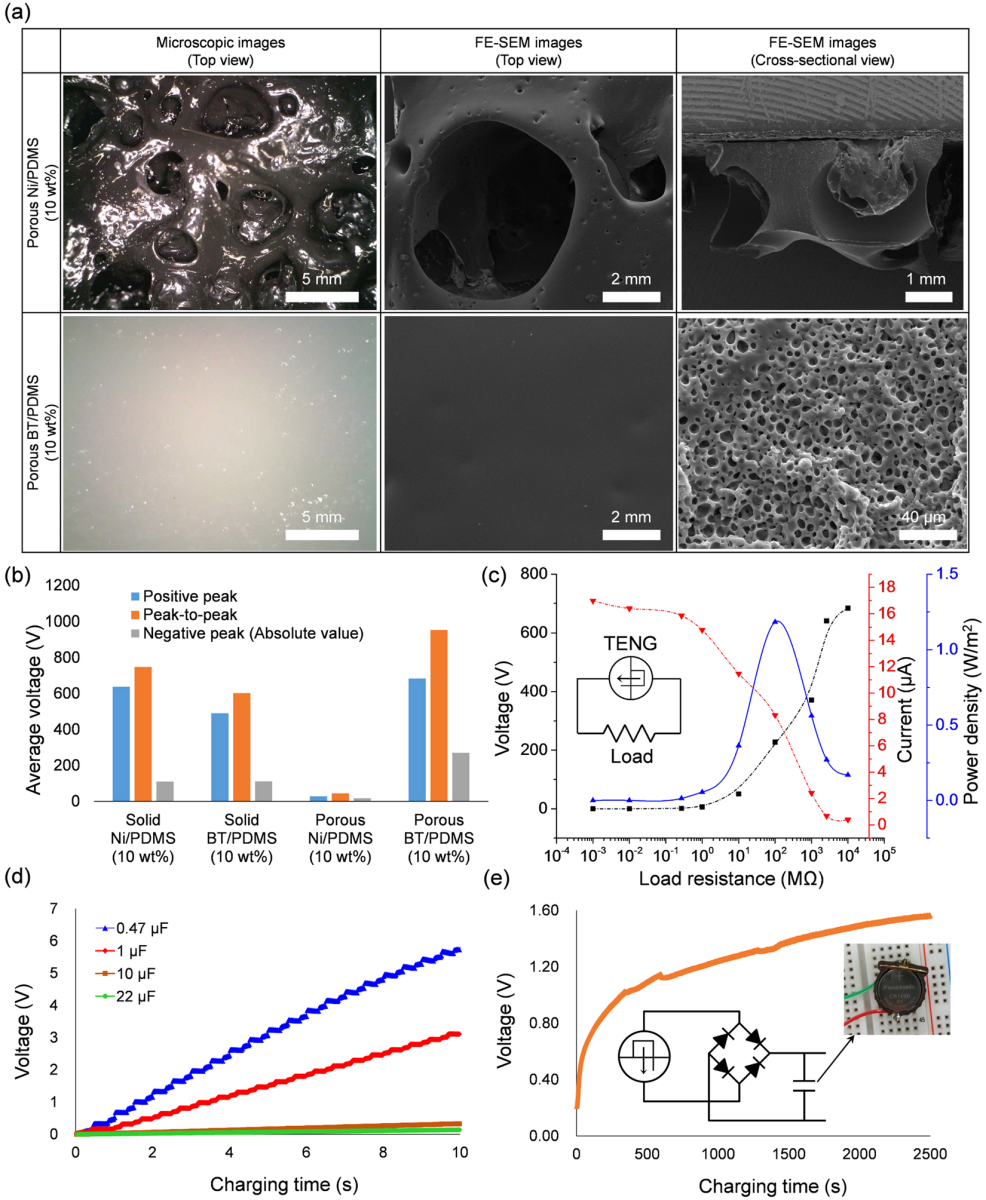
(**a**) Microstructure of porous Ni/PDMS and BT/PDMS nanocomposites. (**b**) Comparison of voltage of TENGs fabricated using the solid and porous nanocomposites. (**c**) Voltage, current and power density as a function of load resistance for the TENG based on porous BT/PDMS nanocomposites. Charging tests of (**d**) various capacitors and (**e**) a lithium coin cell.

Finally, porous Ni/PDMS and porous BT/PDMS nanocomposites were fabricated via MW irradiation. For the precursor, 10 wt% Ni and BT nanoparticles were mixed with the uncured PDMS, respectively. DI water was then added to the mixture with the volume ratio of 4:11 (water to PDMS) and blended again. Once the final precursors were MW irradiated, a completely different porous structure appeared as opposed to the solid PDMS nanocomposites, which is stably fabricated. Severe swelling is observed in the porous Ni/PDMS nanocomposites, similar to the fabrication of porous PDMS using EGC-1720 (see Fig. 5a). Since the Ni has very short penetration depths^26^, the uncured PDMS near the Ni nanoparticles rapidly cures first, and then the water molecules are heated (Fig. S9). At this time, the PDMS becomes rigid. As the residual DI water that has not escaped from the PDMS expands, the PDMS undergoes large swelling and even burst. There are large visible pores at the millimeter level. Meanwhile, micrometer-sized pores were well formed in the BT/PDMS nanocomposites during MW irradiation without such severe swelling. Many micropores were well fabricated and uniformly distributed inside the BT/PDMS. Because the BT is not sensitive to MW, no significant difference from porous PDMS that is fabricated from water and PDMS mixture was observed. The porous nanocomposites were then used in TENG. For the porous Ni/PDMS nanocomposite, the performance of TENG was adversely affected due to the large pores (Fig. 5b). However, the use of porous BT/PDMS improved the positive and negative voltage by more than 83% and 170% compared to pure PDMS, respectively. Incorporating additive nanoparticles such as BaTiO_3_, carbon nanotubes and graphene could enhance the dielectric property (*ε*_*r*_) of the PDMS film and hence improve the electrical performance of TENGs. Thus, porous BT/PDMS nanocomposites fabricated in this study enhances the performance of TENG^18,20^. The current was also measured, and the maximum power density, 1.184 W/m^2^, was obtained at about 100 MΩ load resistance, as shown in Fig. 5c. It should be noted that the power density was obtained from positive peaks. Furthermore, it was confirmed that the TENG based on porous BT/PDMS could not only charge various capacitors and a lithium coin cell but also light up several hundreds of light-emitting diodes (LEDs). A 0.47 μF capacitor was charged up to 5 V within 8 seconds at a frequency of 2 Hz; for 1 μF, 10 μF, and 22 μF capacitors, the charging slopes were 0.3238 V/s, 0.0334 V/s, and 0.014 V/s, respectively (Fig. 5d). Additionally, the lithium coin cell (3 V) was charged up to 1.6 V within 2500 seconds as shown in Fig. 5e. At the same operating condition, 270 blue LEDs were instantaneously turned on by the produced output voltage of TENG fabricated using the porous BT/PDMS nanocomposite (Fig. S10 and Movie S2).

## Conclusion

In summary, a novel methodology through one-step microwave irradiation was proposed to rapidly fabricate porous PDMS nanocomposites without any further post-processing. To construct the porous structure, we selected deionized (DI) water and fluorine solvents with different boiling points as additives. MW irradiation of the additive/PDMS mixture led to remarkably different structures depending on the boiling point; the fluorine solvents with lower boiling points led to severe swelling of the PDMS, while other solvents allowed the porous structure to be well-fabricated. The porous PDMS derived from a water/PDMS mixture showed the best triboelectric performance among the precursor solutions. In addition, porous PDMS nanocomposites were fabricated by adding Ni or BaTiO_3_ nanoparticles into the water/PDMS mixture. Since the Ni nanoparticles rapidly heat up during MW irradiation, a swelling phenomenon was observed, similar to that of the porous PDMS prepared using a mixture of EGC-1720 and PDMS. On the other hand, porous BT/PDMS nanocomposites could be well-fabricated via MW irradiation within a short time, and showed significantly enhanced triboelectric performance. If the proposed concept is well-optimized, it is expected that this methodology can further enhance the performance of TENG. Because MW irradiation can be used in the fabrication of printed conductors in addition to porous elastomeric nanocomposites, the present method would pave the new way for simple, rapid and low-cost fabrication of soft/flexible/stretchable electronics such as on-skin sensors and soft actuators.

## Electronic supplementary material

Supplementary Information

Movie S1

Movie S2
