# Supplementary material for: Rapid Fabrication of Microporous BaTiO3/PDMS Nanocomposites for Triboelectric Nanogenerators through One-step Microwave Irradiation
Source: Sci Rep. 2018 Sep 24;8:14287. doi: 10.1038/s41598-018-32609-6 (PMC6155244; doi:10.1038/s41598-018-32609-6)
Supplement: Supplementary file 1 — Supplementary Information [file 41598_2018_32609_MOESM1_ESM.pdf]

Supplementary Information

Rapid Fabrication of Microporous $BaTiO_3$/PDMS Nanocomposites for Triboelectric Nanogenerators through One-step Microwave Irradiation

Shin Jang and Je Hoon Oh*

Department of Mechanical Engineering, Hanyang University, Ansan, Gyeonggi-do 15588, Republic of Korea

*jehoon@hanyang.ac.kr

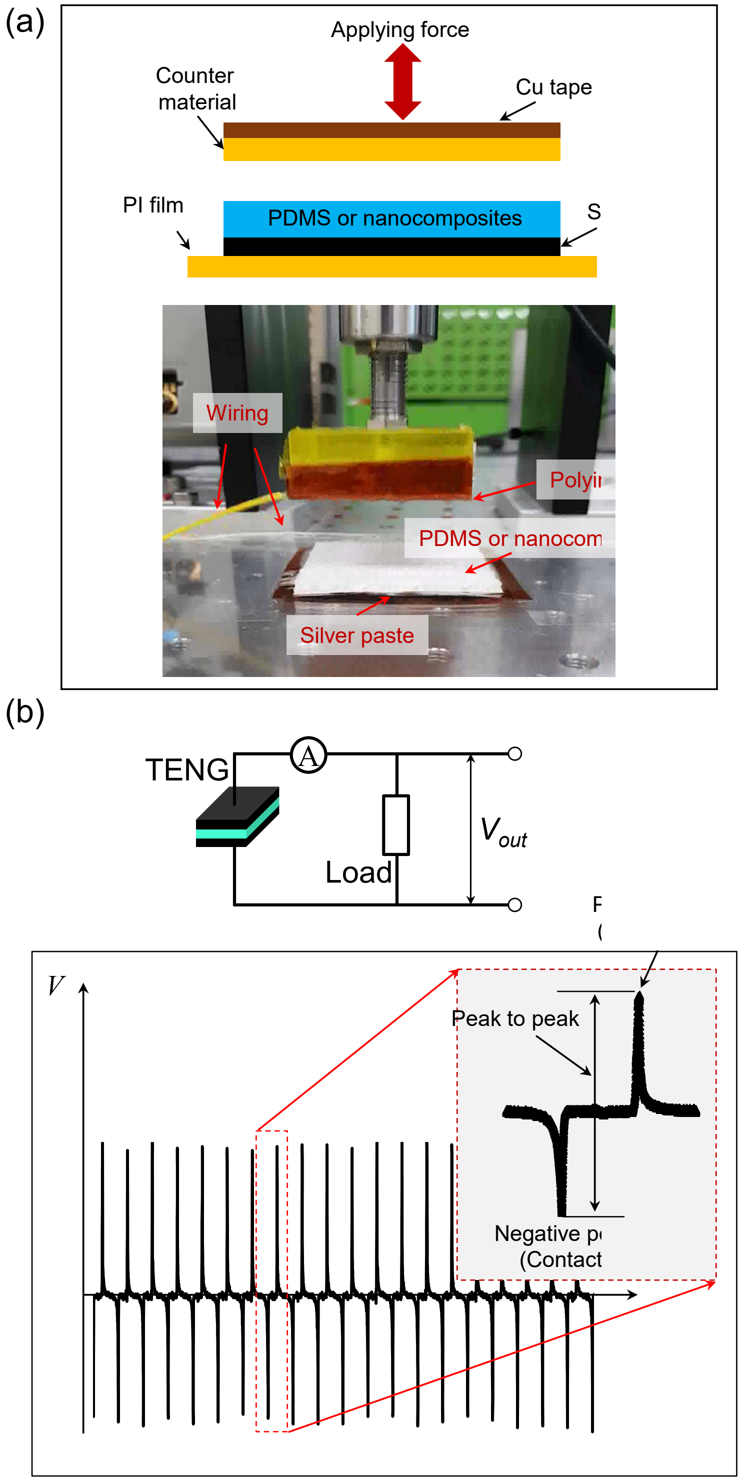

Figure S1. (a) TENG structure and the in-house system for testing. (b) A circuit diagram and voltage output as a function of the contact-separation behavior of the TENG.

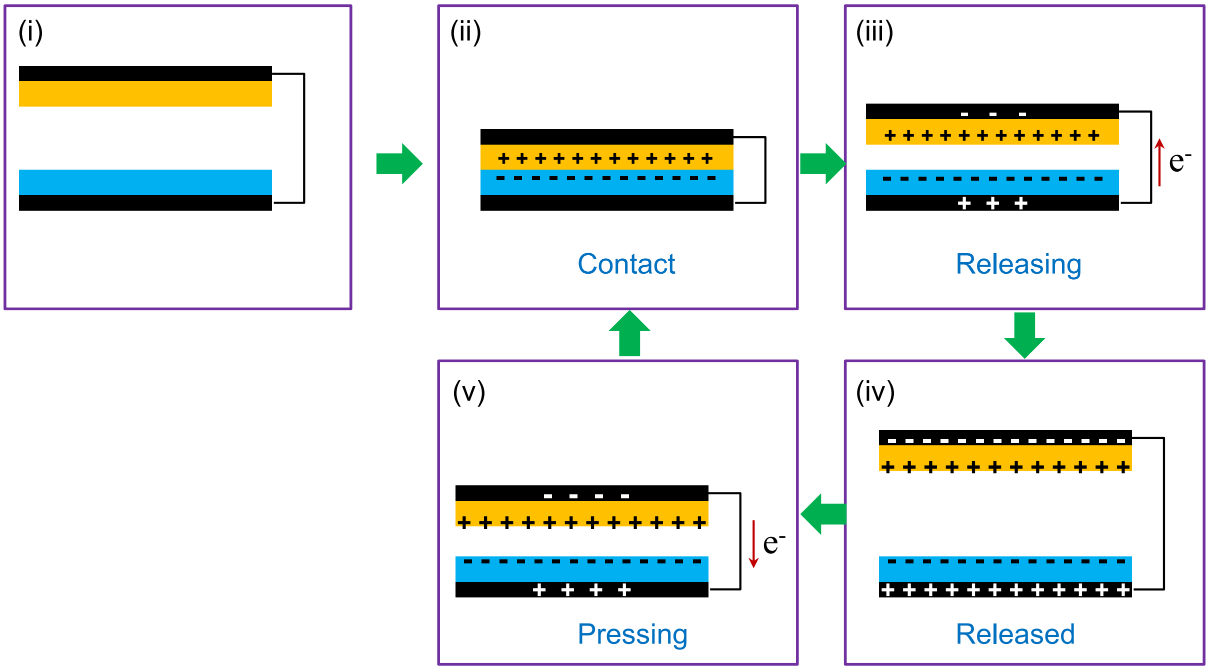

Figure S2. Illustration of the operating mechanism of contact-separation mode TENGs

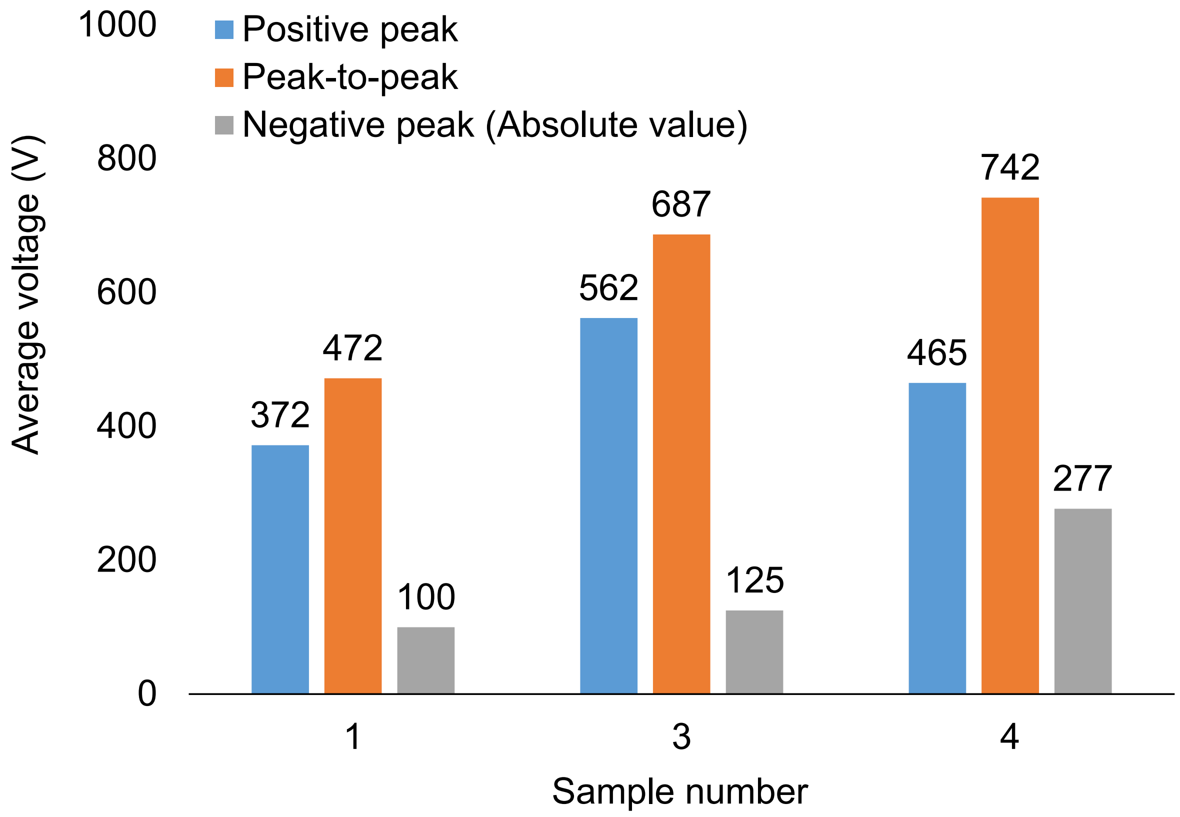

Figure S3. Comparison of output voltage for TENGs fabricated using different solvents.

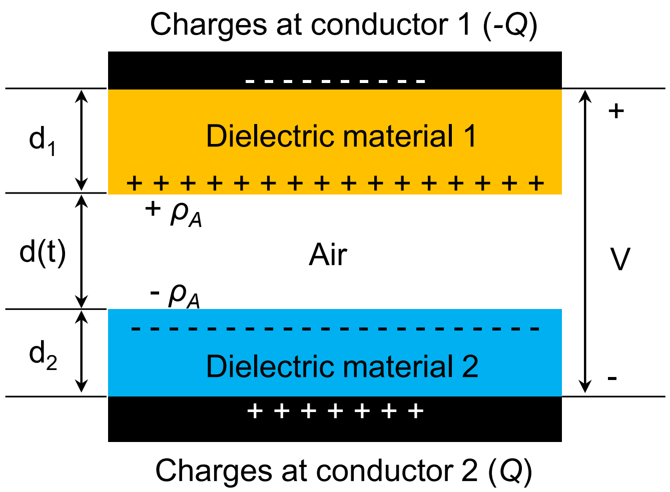

Figure S4. Comparison of output voltage for TENGs fabricated using different solvents.

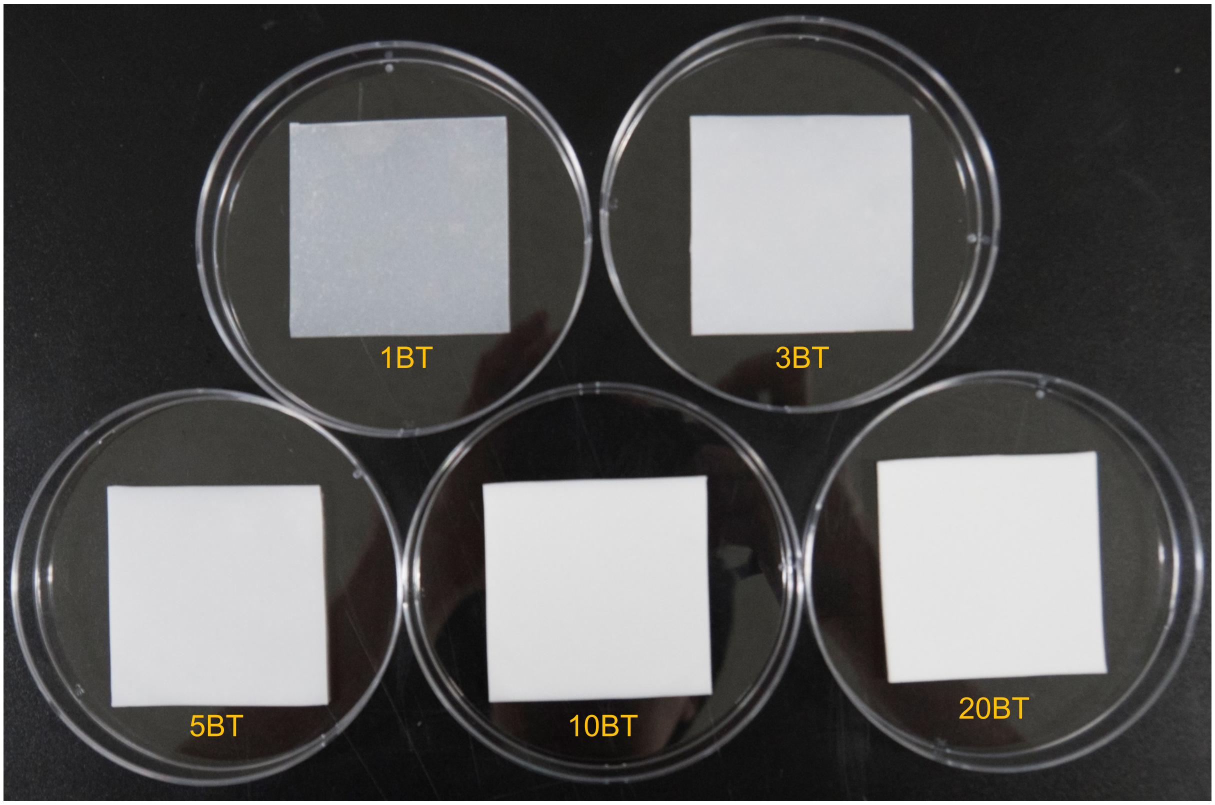

Figure S5. Photographs of solid BT/PDMS nanocomposites fabricated from the BT/PDMS mixture with respect to BT concentration (wt%).

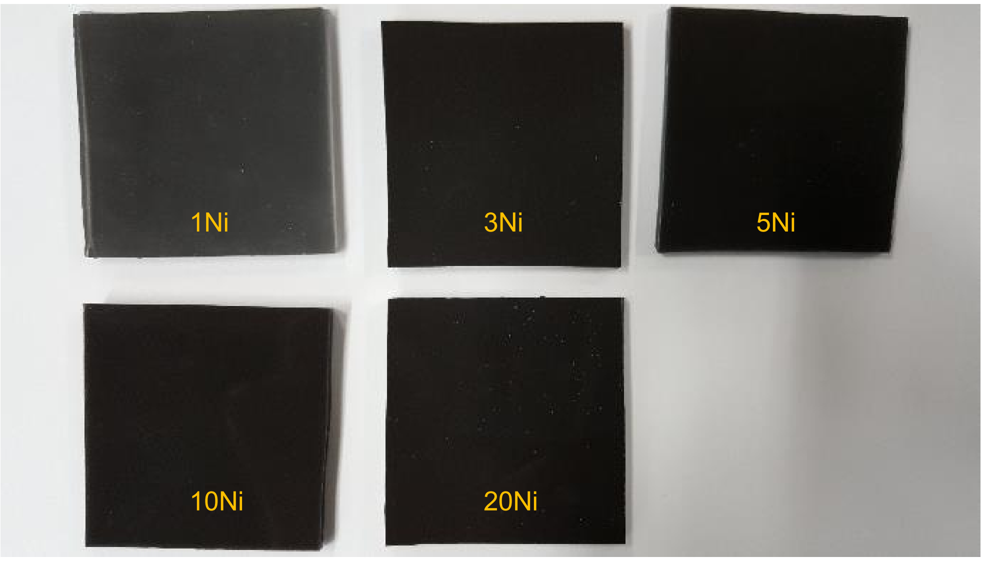

Figure S6. Photographs of solid Ni/PDMS nanocomposites fabricated from the Ni/PDMS mixture with respect to Ni concentration (wt%).

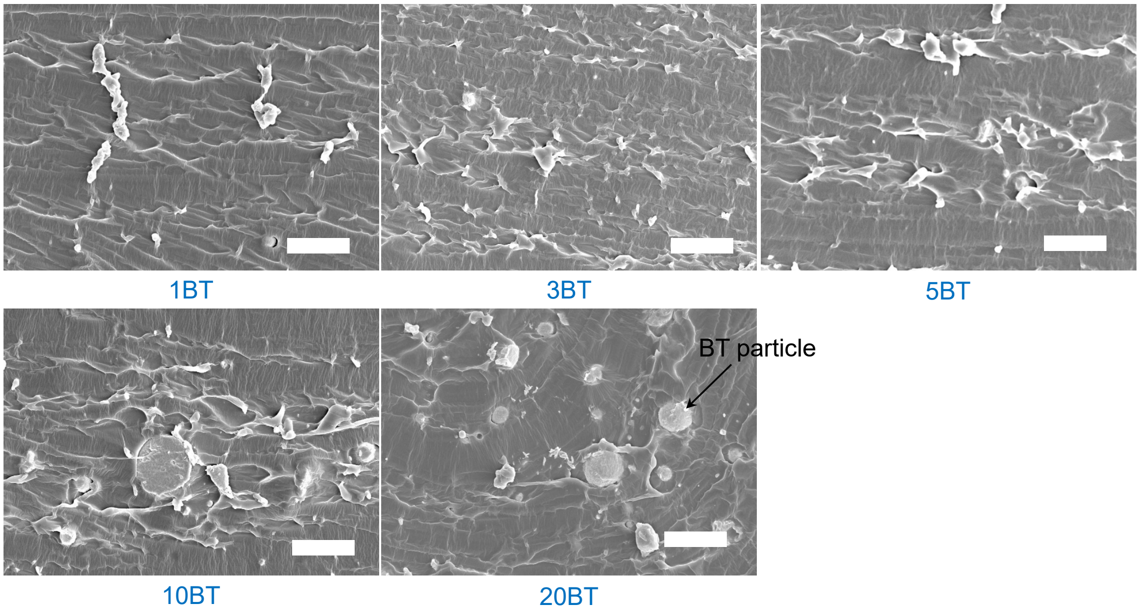

Figure S7. Cross-sectional FE-SEM images of solid BT/PDMS nanocomposites with respect to BT concentration (wt%). Scale bars in the insets are 20 μm.

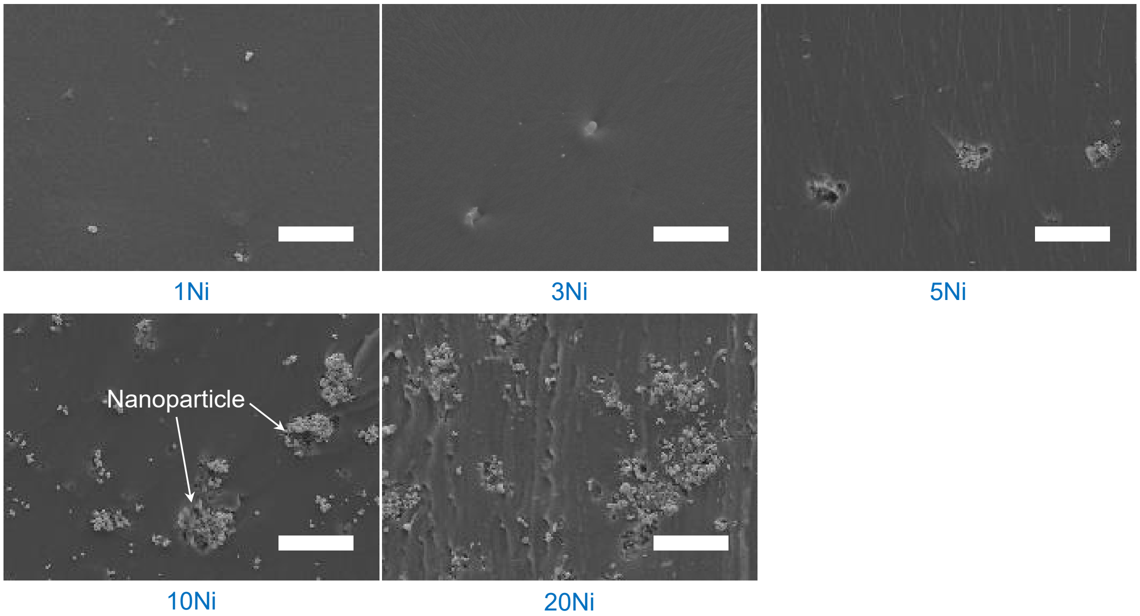

Figure S8. Cross-sectional FE-SEM images of solid Ni/PDMS nanocomposites with respect to Ni concentration (wt%). Scale bars in the insets are 50 μm.

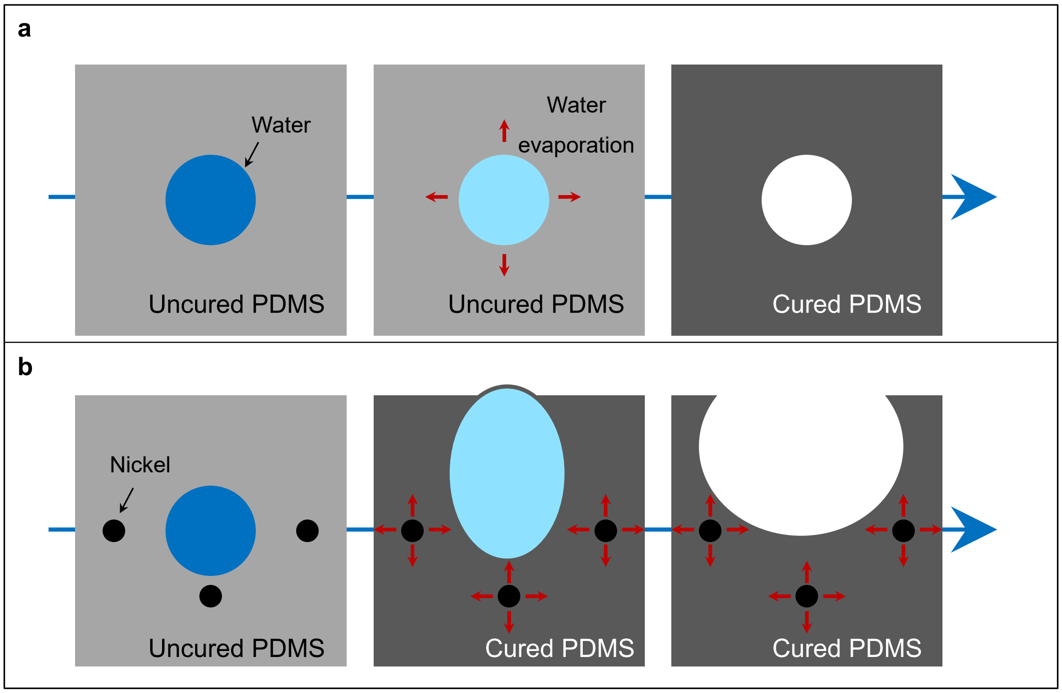

Figure S9. Schematic illustration of swelling mechanism of (a) water/PDMS and (b) water/Ni/PDMS mixtures during MW irradiation.

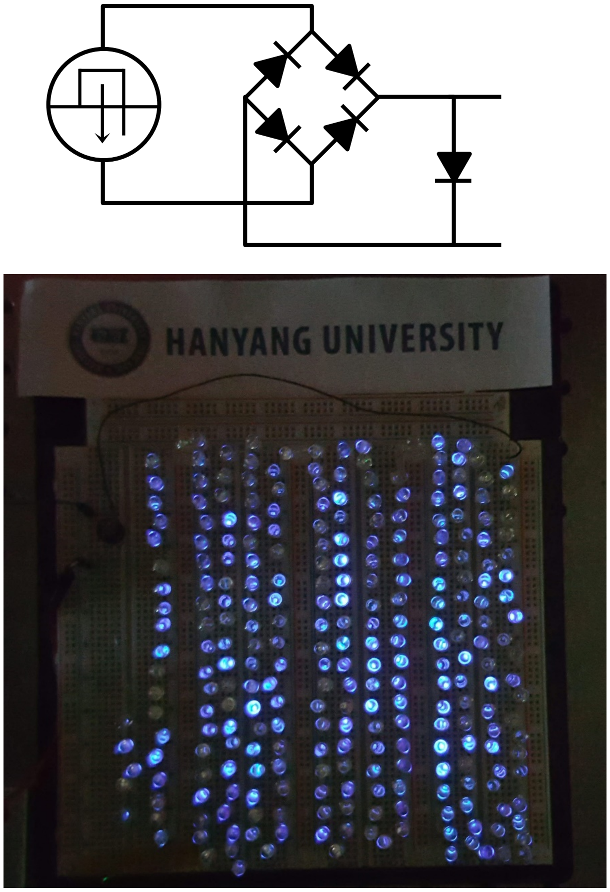

Figure S10. Circuit diagram and a snapshot of LEDs operation.
